# Deep learning for dense Z-spectra reconstruction from CEST images at sparse frequency offsets

**DOI:** 10.3389/fnins.2023.1323131

**Published:** 2024-01-05

**Authors:** Gang Xiao, Xiaolei Zhang, Hanjing Tang, Weipeng Huang, Yaowen Chen, Caiyu Zhuang, Beibei Chen, Lin Yang, Yue Chen, Gen Yan, Renhua Wu

**Affiliations:** ^1^School of Mathematics and Statistics, Hanshan Normal University, Chaozhou, China; ^2^Department of Radiology, Second Affiliated Hospital of Shantou University Medical College, Shantou, China; ^3^College of Engineering, Shantou University, Shantou, China; ^4^Medical Imaging Center, Jieyang People’s Hospital, Jieyang, China; ^5^Department of Radiology, Second Affiliated Hospital of Xiamen Medical College, Xiamen, China

**Keywords:** chemical exchange saturation transfer, deep learning, dense Z-spectra reconstruction, sparse frequency offsets, sequence-to-sequence framework

## Abstract

A direct way to reduce scan time for chemical exchange saturation transfer (CEST)-magnetic resonance imaging (MRI) is to reduce the number of CEST images acquired in experiments. In some scenarios, a sufficient number of CEST images acquired in experiments was needed to estimate parameters for quantitative analysis, and this prolonged the scan time. For that, we aim to develop a general deep-learning framework to reconstruct dense CEST Z-spectra from experimentally acquired images at sparse frequency offsets so as to reduce the number of experimentally acquired CEST images and achieve scan time reduction. The main innovation works are outlined as follows: (1) a general sequence-to-sequence (seq2seq) framework is proposed to reconstruct dense CEST Z-spectra from experimentally acquired images at sparse frequency offsets; (2) we create a training set from wide-ranging simulated Z-spectra instead of experimentally acquired CEST data, overcoming the limitation of the time and labor consumption in manual annotation; (3) a new seq2seq network that is capable of utilizing information from both short-range and long-range is developed to improve reconstruction ability. One of our intentions is to establish a simple and efficient framework, i.e., traditional seq2seq can solve the reconstruction task and obtain satisfactory results. In addition, we propose a new seq2seq network that includes the short- and long-range ability to boost dense CEST Z-spectra reconstruction. The experimental results demonstrate that the considered seq2seq models can accurately reconstruct dense CEST images from experimentally acquired images at 11 frequency offsets so as to reduce the scan time by at least 2/3, and our new seq2seq network contributes to competitive advantage.

## 1 Introduction

Chemical exchange saturation transfer (CEST)-magnetic resonance imaging (MRI) provides a powerful tool to indirectly detect diluted molecules by capturing their saturation transfer to the abundant water pool (Zhou et al., 2003; Cai et al., 2012; Kim et al., 2015; Sun et al., 2016; Zaiss et al., 2018; Li et al., 2020; Cember et al., 2023; Longo et al., 2023). In practice, a sufficient number of two-dimensional (2D) CEST images should be acquired at dense frequency offsets to estimate parameters for quantitative analysis. This comes with the resource-consuming measurement (Zaiss et al., 2018; Li et al., 2020), potentially compromising the viability of the biological sample and imparting high sensitivity to motion artifacts. In some scenarios, the long times of sufficiently sampled CEST images covering the expected offset range limit the CEST-MRI to single-slice acquisitions that may not fully interrogate the spatial heterogeneity of tumors (Randtke et al., 2017) or other organs (Longo et al., 2011), thus limiting the functional information that can be provided (Villano et al., 2021). Therefore, CEST-MRI with a significantly shorter scan time can contribute to a more widespread use. Toward this end, reconstructing dense CEST Z-spectra from 2D acquisitions under a small number of scans can provide a better context to capture and understand biomolecular processes and dynamic patterns accurately.

A way to reduce scan time would be to shorten echo readout. Recent improvements in rapid acquisition focus on echo readout: turbo spin echo (Zhou et al., 2008), gradient and spin echo (Zhu et al., 2010), segmented echo planar imaging approaches (Jones et al., 2012), and gradient echo-based techniques (Khlebnikov et al., 2017; Krishnamoorthy et al., 2017). However, the highly segmented acquisition performed on these approaches often leads to increased blurring (Khlebnikov et al., 2017); in particular, the prior information of the optimized measurement parameters and the chosen data limits their extensive applications (Zaiss et al., 2018). Therefore, the development of computational methods that improve the reconstruction quality of dense CEST images from experimentally acquired images at sparse frequency offsets has the potential to be transformative and can push the capabilities of some imaging-limited CEST-MRI modalities.

Deep learning tools have been used for complicated intelligence for several years and have transformed the analysis and interpretation of complex input data (Alzubaidi et al., 2021; Dong et al., 2021). This has led to several breakthroughs for applications in the field of speech recognition, computer vision, and other applications, allowing researchers to intelligently learn different knowledge and carry out previously unachievable experiments. In particular, deep learning has been used successfully to analyze CEST MRI (Perlman et al., 2022; Cohen and Otazo, 2023; Hunger et al., 2023; Mohammed Ali et al., 2023; Wu et al., 2023). Perlman et al. (2022) proposed a rapid detection method for the early apoptotic responses to oncolytic virotherapy using CEST magnetic resonance fingerprinting and deep learning. To predict multi-pool Lorentzian parameters of CEST-MRI at 7 T, Hunger et al. (2023) developed a deepCEST approach by inputting uncorrected Z-spectra of a single B1 level and a B1 map, thereby achieving an uncertainty quantification in addition to the Lorentzian amplitudes. To enhance the super-resolution performance of CEST-MRI, Wu et al. (2023) developed a Cross-space Optimization-based Mutual learning network by incorporating novel spatio-frequency extraction modules and a mutual learning module.

To that end, this study aims to establish a sequence-to-sequence (seq2seq) framework to reconstruct dense CEST Z-spectra from experimentally acquired images at sparse frequency offsets. The main benefit of our seq2seq framework is that it is both simple and efficient when we apply it to tackle the problem of dense Z-spectra reconstruction. Another task of this study is to boost dense Z-spectra reconstruction. In fact, CEST Z-spectra behave like the short- and long-term memory when an MRI scan is performed over the offset frequency range. Surprisingly, the temporal convolutional network (TCN) (Bai et al., 2018) that enhances the capacity of mining higher-level spatial features from historical data and the long short-term memory (LSTM) network (Sherstinsky, 2020) that better remembers the connections in the short time scale of the data can achieve the purpose of learning complex interactions more effectively for the input sequence. Therefore, this paper formulates a TCN-LSTM network to improve reconstruction ability.

## 2 Materials and methods

### 2.1 *In vivo* MRI experiments

The *in vivo* CEST-MRI was performed on a 7.0 T horizontal bore small animal MRI scanner (Agilent Technologies, Santa Clara, CA, United States), with a surface coil (Time Medical Technologies, China) for transmission and reception. For this assessment, 8-week-old male SD rats (Beijing Vital River Laboratory Animal Technology Co., Ltd.) weighing 250 g were used to generate animal models. All animal care and experimental procedures were in accordance with the National Research Council’s Guide for the Care and Use of Laboratory Animals.

In the experimental study, the rat was anesthetized with isoflurane mixed with O_2_ at the rate of 1 L/min; for anesthesia induction, 4.0% isoflurane was used and 2.0%–3.0% isoflurane was used for maintenance. The respiratory probe was used to monitor the breath rate throughout the MRI experiments. The respiration rate and body temperature during the scan in 7 T were 60–70 times/min and 38.5–39.5°C, respectively. Prior B_0_ shimming was used to eliminate signal interference of the B_0_ inhomogeneity in the experiments. Imaging parameters were as follows: repetition time (TR) = 6,000 ms, echo time (TE) = 40 ms, array = frequency offsets, slice thickness = 2 mm, field of view (FOV) = 64 × 64 mm, matrix size = 64 × 64, spatial resolution = 1× 1 mm, and averages = 1. An echo planar imaging (EPI) readout sequence was used to obtain CEST images where continuous wave (CW) RF irradiation was implemented on scanners. Some parameters of the EPI sequence are saturation power = 1.2 μT, RF pulse duration = 1,500 ms, pulse shape = rectangular RF pulse, and shot number = single shot.

The experimentally acquired CEST images at 11 frequency offsets *F* = [−6.00, −3.60, −2.64, −1.68, −1.20, 0.96, 1.92, 2.76, 3.36, 3.96, and 6.00] ppm were the inputs of considered reconstruction models. The experimentally acquired CEST images at 101 frequency offsets evenly distributed from −6 to 6 ppm were used to evaluate the performance of the reconstruction model by comparing the estimates with the experimental data values (ground truth).

### 2.2 Training dataset

In this study, Z-spectra simulations were performed to train the considered networks, solving the labor-intensive and time-consuming task of obtaining labeled training data from actual experiments. Simulated CEST Z-spectra at non-uniform sparse frequency offsets and uniform dense frequency offsets were obtained using a 7-pool Bloch–McConnell equation (Xiao et al., 2023). This 7-pool model consists of free water centered at 0 ppm, amide centered at 3.5 ppm, guanidyl/amine centered at 2.0 ppm, hydroxyl centered at 1.3 ppm, nuclear Overhauser enhancement (NOE) centered at −1.6 ppm, magnetization transfer (MT) centered at −2.4 ppm, and NOE centered at −3.5 ppm.

The multi-pool properties of *in vivo* Bloch–McConnell simulations are listed in Table 1 (Tang et al., 2023). In total, 20 dynamic parameters were considered for tissue combinations. For each parameter, their variables were randomly sampled in a uniform distribution between the lower bound (LB) and upper bound (UB). In practice, the sampled variables of each parameter interacting with that of each other generated 350,000 parameter combinations, thus yielding 350,000 simulated Z-spectra (Supplementary Figure S1).

**Table 1:** Summary of the parameters in generating simulated data: solute concentration (*f*_s_), solute-water exchange rate (*k*_sw_), longitudinal relaxation time (*T*_1_), transverse relaxation time (*T*_2_), and solute resonance frequency offset (Δ).

		*f_s_*(10^−3^)	*k_sw_*(s^−1^)	*T*_1_(s)	*T*_2_(ms)	Δ(ppm)
Amide	LB ~ UB	0.1 ~ 3	1 ~ 100	1 ~ 1	0.5 ~ 100	3.5
Guanidyl/Amine		0.1 ~ 2	100 ~ 1,000	1 ~ 1	0.1 ~ 5	2
Hydroxyl		0.1 ~ 10	100 ~ 2000	1 ~ 1	0.1 ~ 5	1.3
Free water		1,000 ~ 1,000	–	1 ~ 3	20 ~ 100	0
NOE (−1.6)		1 ~ 5	1 ~ 50	1 ~ 1	0.1 ~ 1	−1.6
MT		10 ~ 200	1 ~ 50	1 ~ 1	0.01 ~ 0.1	−2.4
NOE (−3.5)		10 ~ 50	1 ~ 40	1 ~ 1	1 ~ 10	−3.5

### 2.3 Training process

In the training process, 50 epochs and a batch size of 512 were used for training; the initial learning rate was 0.001, the learning rate was halved every five epochs, and the random deactivation ratio was 0.2. Adam was used as the training optimizer, and RELU was the activation function. The 350,000 paired CEST data obtained by the Bloch-McConnell equation were divided into training and validation sets in a ratio of 7:3, with data at sparse frequency offsets as input and data at dense frequency offsets as target. The CEST images at sparse and dense frequency offsets obtained from the actual MRI experiments were used as the test set. The experimentally acquired CEST images at sparse frequency offsets were input to the trained seq2seq model to reconstruct the dense CEST images, and the reconstructed data were compared with the ground-truth (experimentally acquired CEST images at dense frequency offsets) to evaluate the performance of considered models.

### 2.4 Evaluation metrics and workstation

To evaluate the proposed model in reconstructing Z-spectra for each pixel of CEST images, the absolute error modulus, the regress analysis (Nagelkerke, 1991), the structural similarity index (SSIM), and the peak signal-to-noise ratio (PSNR) (Hore and Ziou, 2010) were applied to evaluate the reconstruction performance of the proposed model and its counterparts.

The workstation used in this study was a Dell T7810 workstation with 16 G memory, dual-core CPU12 core, and a 3.4 G main operating frequency. The experiments were based on PyTorch. We initialized the weights using samples from a uniform distribution *N*(0,0.01) and used the MSE-Loss as the loss function.

### 2.5 Sequence-to-sequence model for reconstructing dense Z-spectra

To reconstruct dense CEST Z-spectra by the seq2seq models, recurrent neural network (RNN) (Sherstinsky, 2020), LSTM (Sherstinsky, 2020), gate recurrent unit (GRU) (Dey and Salem, 2017), TCN (Bai et al., 2018), and the proposed TCN-LSTM are considered. In addition, the multiple-pool Lorentzian fitting (Cui et al., 2022) is included in comparison experiments, as described in Supplementary material S1. The introduction of RNN, LSTM, and GRU is presented in Supplementary material S2. The principle of TCN and TCN-LSTM network is briefly described in Supplementary material S3.

**Figure 1:**
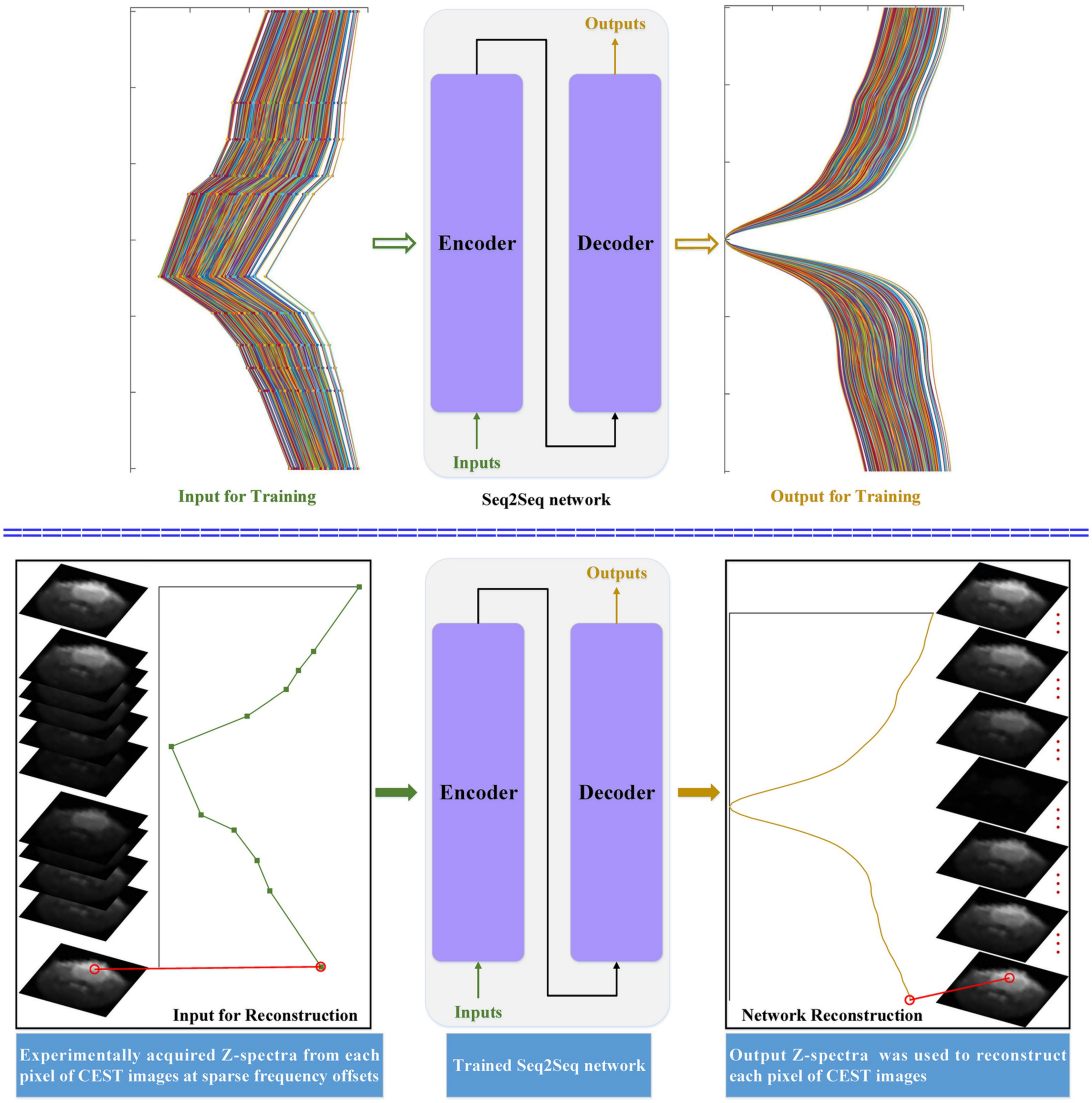
The flowchart of the developed seq2seq framework when it is applied to reconstruct dense CEST Z-spectra from experimentally acquired images at sparse frequency offsets.

Figure 1 depicts the flowchart of the considered seq2seq models when they are applied to reconstruct dense Z-spectra and dense CEST images. The simulated Z-spectra are generated by using the Bloch–McConnell equations. Simulated Z-spectra at sparse and dense frequency offsets are used to train the seq2seq network. The main intuition behind simulated data is that we can generate as much data as needed with all possible tissue combinations to train a seq2seq network. Then, the trained seq2seq network is used to reconstruct dense Z-spectra from experimentally acquired Z-spectra for each pixel of CEST images at sparse frequency offsets. The sequence-to-imaging reconstruction for each pixel of dense CEST images is generated by the output Z-spectra.

## 3 Results

### 3.1 CEST Z-spectra reconstruction

**Figure 2:**
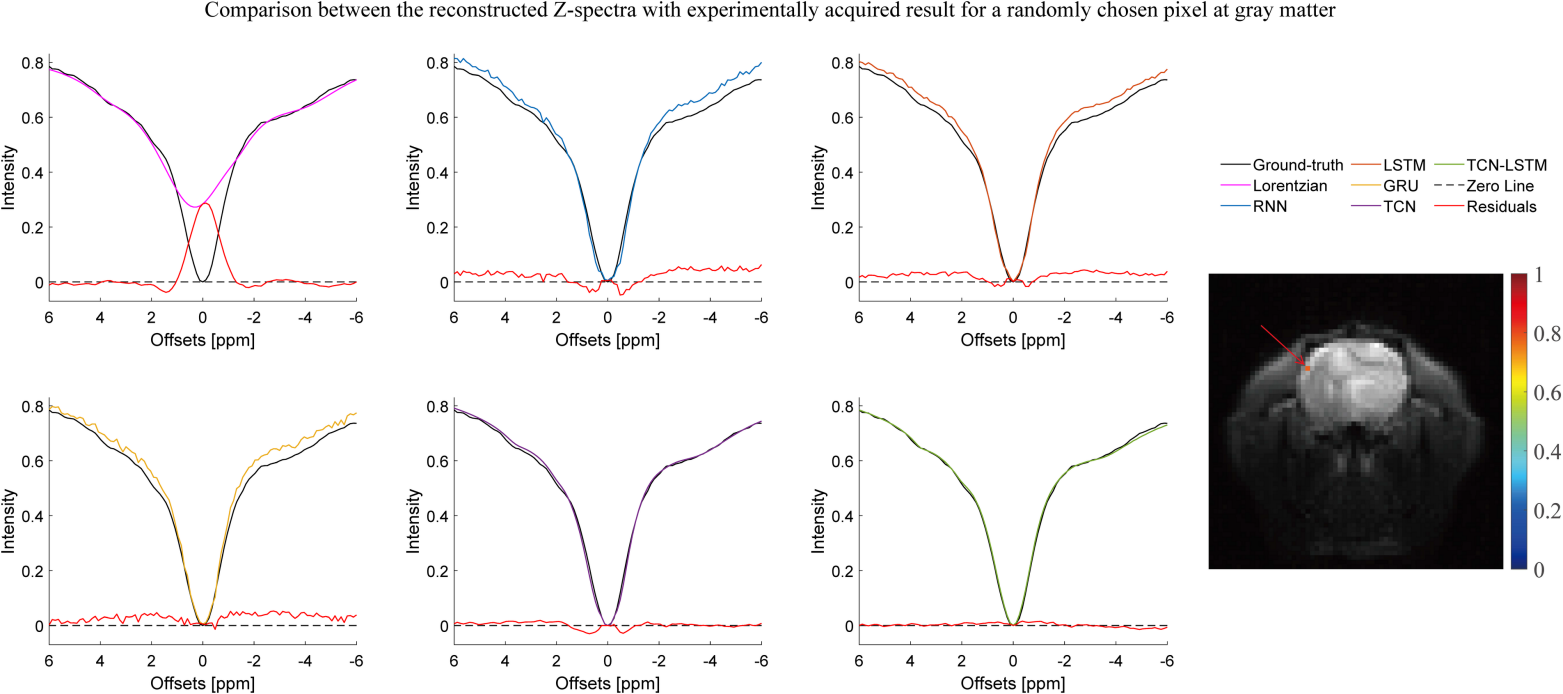
Comparison between the reconstructed Z-spectra with the experimentally acquired result for a randomly chosen pixel at gray matter.

**Figure 3:**
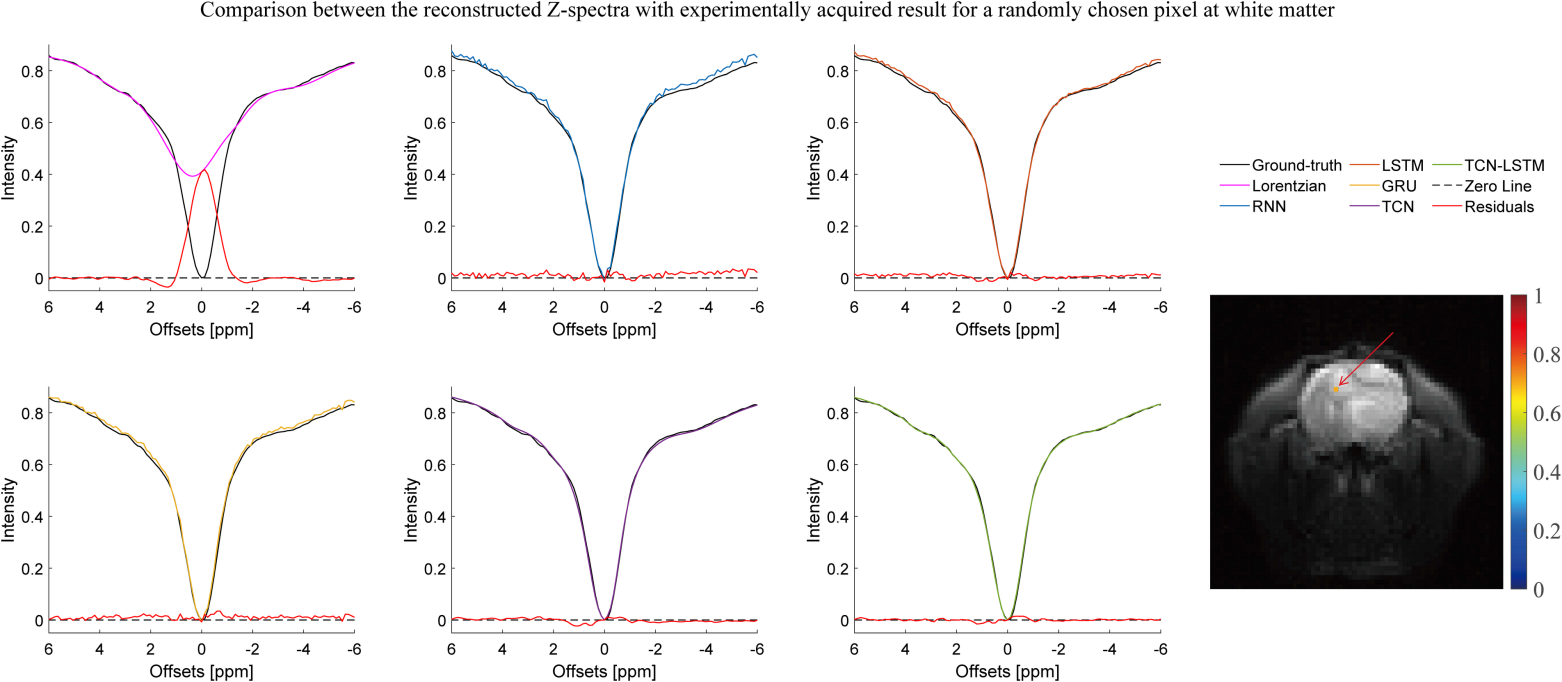
Comparison between the reconstructed Z-spectra with the experimentally acquired result for a randomly chosen pixel at white matter.

**Figure 4:**
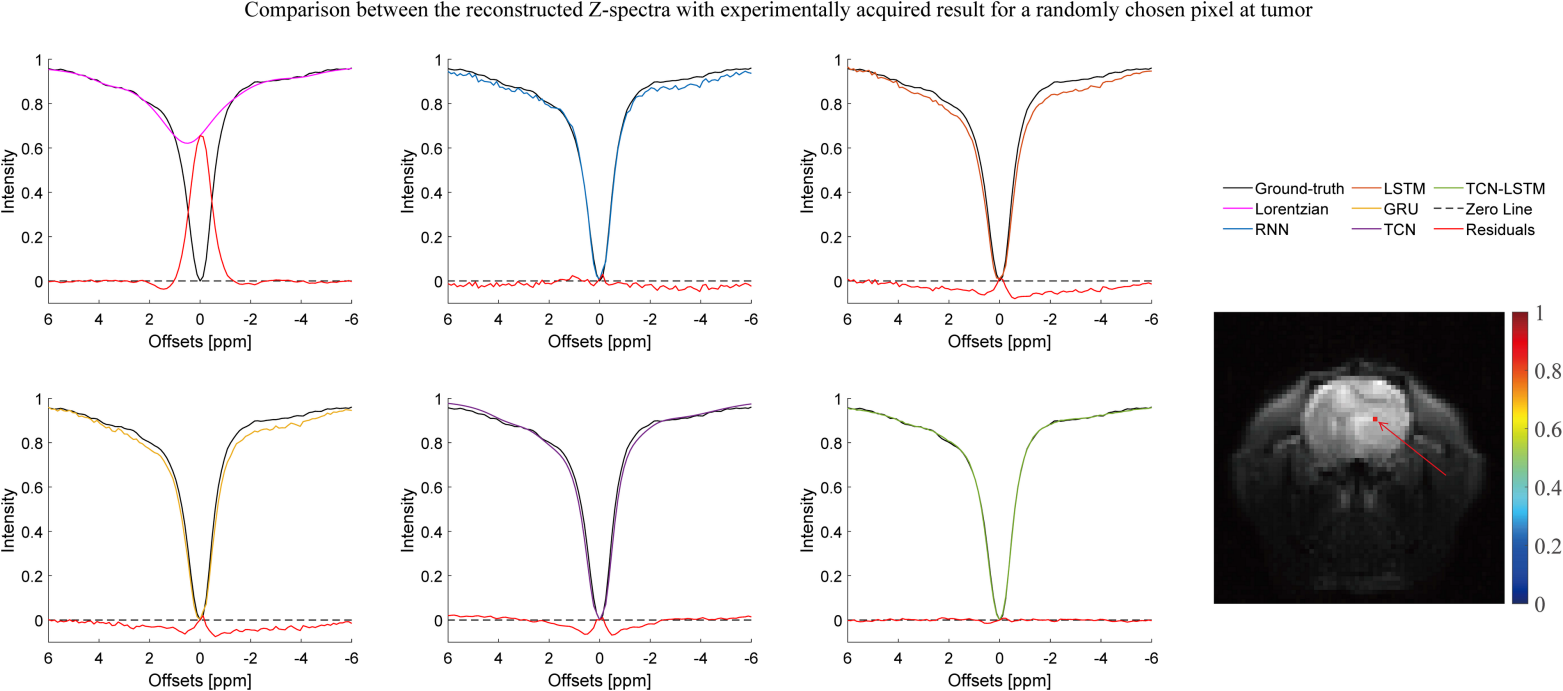
Comparison between the reconstructed Z-spectra with the experimentally acquired result for a randomly chosen pixel at the tumor.

Figures 2–4 present the reconstructed Z-spectra of each model for a randomly chosen pixel at white matter, gray matter, and tumor, respectively. The multiple-pool Lorentzian fitting demonstrates good consistency when they reconstruct Z-spectra at the known input frequency offsets, while it shows high volatility or shift with the Z-spectral points between [−2 ppm and 2 ppm]. In contrast, RNN, LSTM, and GRU achieve high accuracy and consistency, when they reconstruct the Z-spectra points around 0 ppm, but the curves of reconstructed Z-spectra deviate from the ground truth over other frequency ranges. Note that the TCN-LSTM model displayed a great agreement with the ground truth around turning points, following the same tendency of actual measurements.

### 3.2 CEST image reconstruction

**Figure 5:**
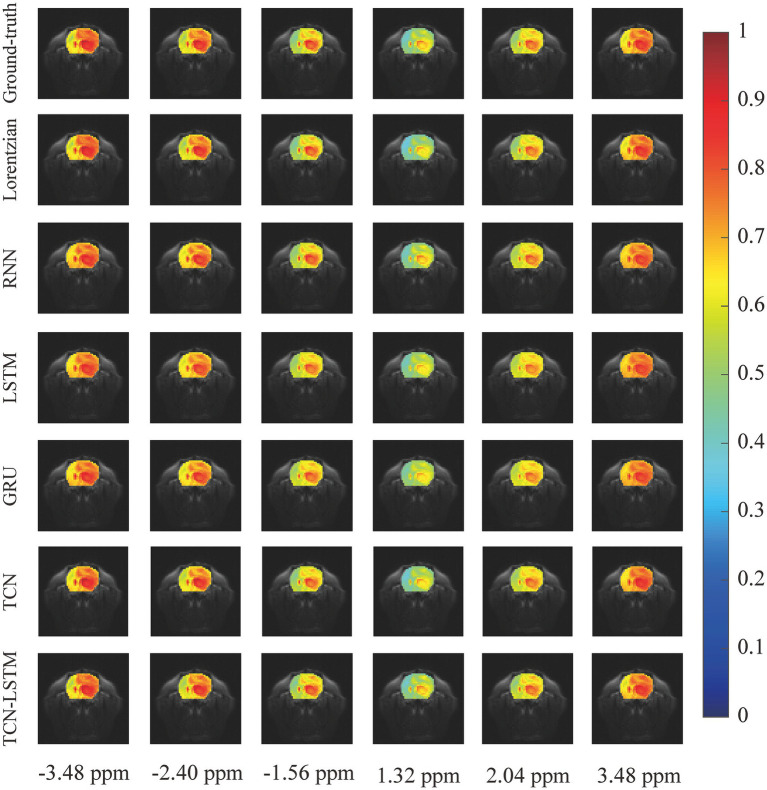
The reconstructed CEST images at frequency offsets −3.48 ppm, −2.40 ppm, −1.56 ppm, 1.30 ppm, 2.04 ppm, and 3.48 ppm when the considered methods are applied to reconstruct dense CEST images from experimentally acquired images at sparse frequency offsets.

**Figure 6:**
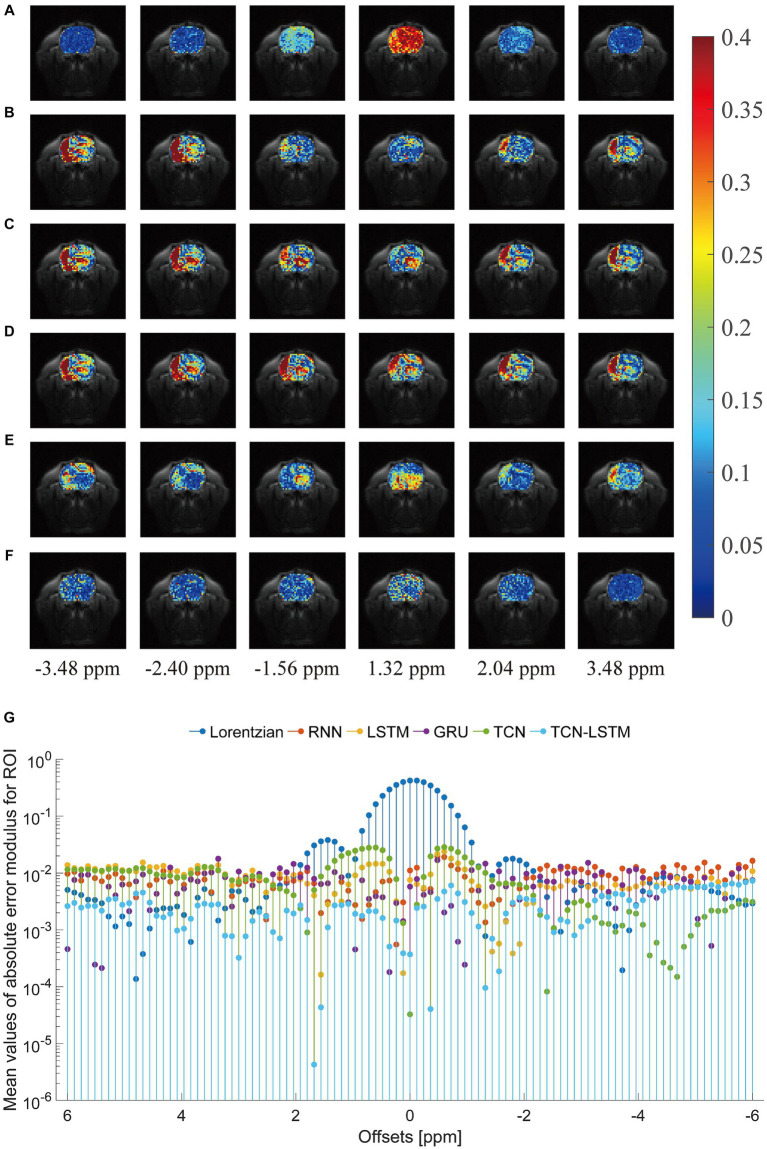
Comparisons of the reconstructed CEST images with the ground-truth. **(A–F)** indicate the absolute error modulus of Lorentzian, RNN, LSTM, GRU, TCN and TCN-LSTM when they are compared with ground-truth at frequency offsets −3.48 ppm, −2.40 ppm, −1.56 ppm, 1.30 ppm, 2.04 ppm and 3.48 ppm. **(G)** The mean values of absolute error modulus across each offset (−6~6 ppm).

To investigate the potential for the considered methods to accurately reconstruct CEST images, we initially conducted an experiment at frequency offsets −3.48 ppm, −2.40 ppm, −1.56 ppm, 1.30 ppm, 2.04 ppm, and 3.48 ppm, as shown in Figure 5. The region of the pseudo-color image overlaid on the anatomy image was the region of interest (ROI). From this figure, the reconstructed results obtained by the considered methods were almost identical to the ground truth. Considering the limited visualization by the naked eye, we further compared these methods using other evaluation metrics, as shown in Figure 6. In Figures 6A–F were the absolute error modulus of Lorentzian, RNN, LSTM, GRU, TCN, and TCN-LSTM when they were compared with the experimentally acquired CEST images (ground-truth), respectively. It was clear that the strength of each method strategy was sufficient to reconstruct patterns at these offsets. As a next step, Figure 6G depicted the mean values of absolute error modulus when the considered models were applied to CEST images at each offset (−6 ~ 6 ppm) for ROI. There were large deviations between the Lorentzian fitting and the ground truth in the range of [−2 ppm and 2 ppm], while RNN, LSTM, GRU, TCN, and TCN-LSTM gave satisfactory results along each frequency offset.

In practice, the smaller values of absolute error modulus indicate better visual quality, yet no standards of CEST image quality have been published. From Figures 5, 6G, the threshold of absolute error modulus below 3% should be acceptable if we consider both subjective experience and objective factors.

### 3.3 Statistical analysis

**Figure 7:**
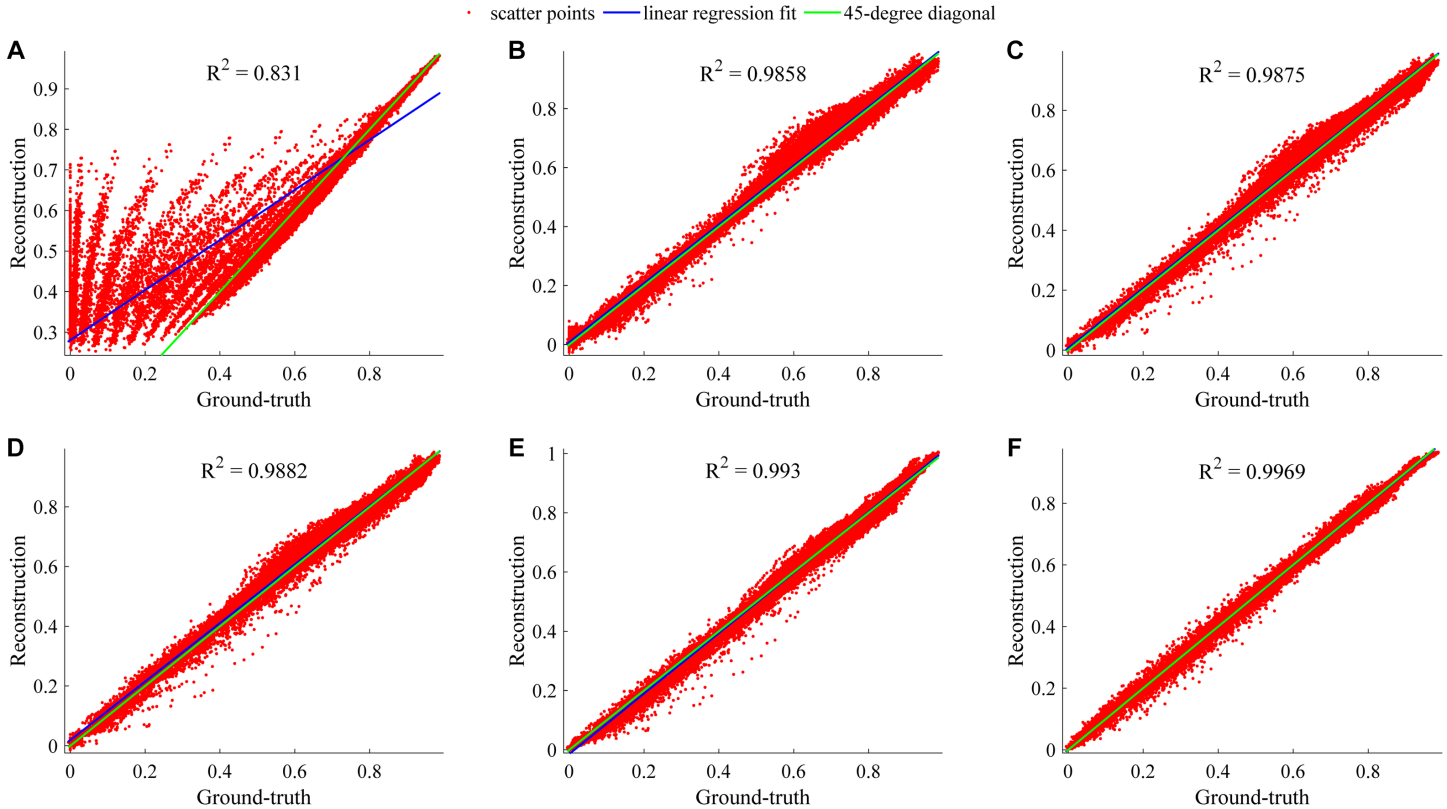
Regression analysis of considered methods when they are applied to reconstruct dense CEST images from experimentally acquired images at sparse frequency offsets. **(A–F)** Denote the results of Lorentzian, RNN, LSTM, GRU, TCN, and TCN-LSTM, respectively.

The regression analysis on the ROI-based whole data of reconstructed CEST images at 101 frequency offsets was designed to assess the general performances of our TCN-LSTM and its counterparts. Figure 7 displays the linear regression plots obtained from the different models tested in this analysis. Note that the coefficient of determination *R*^2^ denotes the proportion of the variability that can be attributed to its linear relation with the ground truth. It can be observed that RNN, LSTM, GRU, TCN, and TCN-LSTM perform well in this analysis (*R*^2^ > 0.985), and the results support the observation that the reconstructions for the whole data seem to be fully consistent with the ground truth. The method based on TCN-LSTM presents a better agreement between its reconstruction and the ground truth (*R*^2^ = 0.9969). It should be noted that the multiple-pool Lorentzian fitting provides a lower coefficient of determination and the scatters deviate from the 45-degree diagonal because Lorentzian fitting can only reconstruct Z-spectra at the known input frequency offsets.

### 3.4 CEST image quality assessment

**Figure 8:**
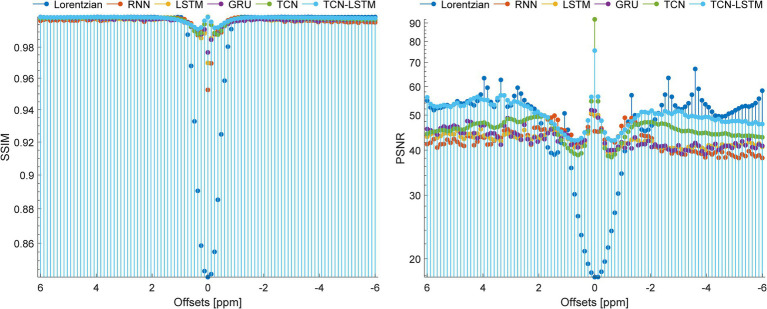
SSIM and PSNR of considered methods when they are applied to reconstruct dense CEST images from experimentally acquired images at sparse frequency offsets.

To set up a comprehensive way to evaluate the performance of different methods, Figure 8 demonstrates the SSIM and PSNR from the ground truth and the reconstruction at each offset (−6 ~ 6 ppm). Table 2 lists the mean values of SSIM and PSNR obtained by considered methods. Clearly, our model exhibits competitive results at each offset in terms of these two metrics, and other methods are good at both SSIM and PSNR.

**Table 2:** Mean values of SSIM and PSNR obtained by the considered methods when they are applied to reconstruct dense Z-spectra from experimentally acquired images at 11 frequency offsets.

	Lorentzian	RNN	LSTM	GRU	TCN	TCN-LSTM
SSIM	0.9875	0.9967	0.9971	0.9973	0.9984	0.9989
PSNR	47.7917	42.5169	42.6597	43.0040	45.8753	50.3227

## 4 Discussion

As mentioned above, we know that reducing scan time while preserving image quality is crucial in CEST-MRI. In fact, due to the physical limitation of an MRI scanner, it is difficult to juggle scan time and CEST image quality in some scenarios. In this paper, we implemented seq2seq as a novel model to provide dense Z-spectra reconstruction for each pixel of CEST images from experimentally acquired CEST images at sparse frequency offsets. In the experimental analysis, the effectiveness of the multiple-pool Lorentzian fitting and the seq2seq models in terms of the absolute error modulus, the regression analysis, the SSIM, and the PNSR are validated. It should be noted that the case studies of this paper were performed with B_1_ < 2μT. Since our simulated Z-spectra were generated using the Bloch–McConnell equation, the proposed reconstruction model can be extended to other B_1_ fields by adjusting pool size.

In fact, CEST Z-spectra is characterized as a process of short- and long-term memory (Supplementary Figure S2). Fortunately, the TCN-LSTM can capture the short-long correlation of time series by combining TCN and LSTM, achieving the purpose of obtaining the complete propagation information of each point for the input sequence. By training the TCN-LSTM, we can solve the inverse problem of inferring the parameters of interest from the measured data. Consequently, the TCN-LSTM’s potential in a wide range of missing data problems can be even greater than the multiple-pool Lorentzian fitting and other seq2seq models (Figures 2–4). By that, the performance of TCN-LSTM in terms of regression analysis between the reconstruction and the ground truth is surprising, revealing the highest coefficient of determination (Figure 7). In image quality assessment, the TCN-LSTM consistently provided better performances in terms of the SSIM and PSNR (Figure 8; Table 2).

Also, the training of deep networks for predicting objects requires a large dataset with high quality, which is extremely expensive to construct in experiments. In fact, the measured Z-spectra can be derived by solutions of the Bloch–McConnell equations, as presented in this study. To solve this problem, we built an automatically labeled dataset based on our designed Bloch–McConnell equations by considering various parameters in a wide range (Table 1). This is a notable advantage of our simulator because the accuracy of the reconstruction is no longer strictly limited by the sample size and experimental conditions, allowing improvement in the efficiency and quality of training. With that, our CEST simulator successfully implemented the generation of the training dataset, and the experimental results proved its superiority.

## 5 Conclusion

In this paper, the authors developed a seq2seq framework to reconstruct high-quality dense Z-spectra for each pixel of CEST images from experimentally acquired CEST images at sparse frequency offsets, demonstrating that general dynamics can be learned from simulated scenes and applied to CEST datasets acquired in experiments. The developed seq2seq framework can achieve the reconstruction of dense CEST images simultaneously only by inputting a Z-spectra for each pixel, allowing scan time reduction and solving the task of high-quality CEST data reconstruction.

## Data availability statement

The original contributions presented in the study are included in the article/Supplementary material, further inquiries can be directed to the corresponding authors.

## Ethics statement

The animal studies were approved by National Research Council’s Guide for the Care and Use of Laboratory Animals. The studies were conducted in accordance with the local legislation and institutional requirements. Written informed consent was obtained from the owners for the participation of their animals in this study.

## Author contributions

GX: Conceptualization, Methodology, Software, Writing – review & editing. XZ: Conceptualization, Formal analysis, Methodology, Visualization, Writing – original draft, Writing – review & editing. HT: Conceptualization, Software, Visualization, Writing – original draft. WH: Formal analysis, Investigation, Validation, Writing – review & editing. YaC: Investigation, Project administration, Supervision, Writing – review & editing. CZ: Data curation, Investigation, Resources, Writing – original draft. BC: Resources, Validation, Writing – original draft. LY: Investigation, Resources, Writing – original draft. YuC: Data curation, Resources, Writing – original draft. GY: Formal analysis, Investigation, Supervision, Validation, Writing – review & editing. RW: Funding acquisition, Investigation, Project administration, Supervision, Writing – review & editing.
