# Supplementary material for: Deep learning for dense Z-spectra reconstruction from CEST images at sparse frequency offsets
Source: Front Neurosci. 2024 Jan 5;17:1323131. doi: 10.3389/fnins.2023.1323131 (PMC10796656; doi:10.3389/fnins.2023.1323131)
Supplement: Supplementary file 1 [file Data_Sheet_1.PDF]

Supporting Material FILE S1: Multiple-pool Lorentzian fitting.

The multiple-pool Lorentzian fitting of Z spectra is performed using a non-linear optimization algorithm:

$$S(\Delta\omega)/S_0 = 1 - \sum_{i=1}^{N} L_i(\Delta\omega) \tag{1}$$

where

$$L_i(\Delta\omega) = \frac{A_i}{1 + \frac{(\Delta\omega - \Delta_i)^2}{(0.5W_i)^2}} \tag{2}$$

Eq. (2) represents a Lorentzian line with central frequency offset from water (Δ_i), peak FWHM (W_i), and peak amplitude (A_i). The value of N is the number of fitted pools; S is the measured signal on the Z-spectra; and S_0 is the non-irradiation control signal.

In this study, a seven-pool model Lorentzian fit including Amide at 3.5 ppm (L_1), Guanidyl/Amine at 2.0 ppm (L_2), Hydroxyl at 1.3 ppm (L_3), Water at 0 ppm (L_4), NOE at −1.6 ppm (L_5), NOE at −3.5 ppm (L_6), and MT at -2.4 ppm (L_7) was performed to process the Z-spectra.

Supporting Material FILE S2: RNN, LSTM and GRU networks.

The RNN is the general class of a neural network that possesses internal memory, enabling it to capture sequential dependencies. Unlike traditional neural networks that treat inputs as independent entities, RNNs consider the temporal order of inputs, making them suitable for tasks involving sequential information. By employing a loop, RNNs apply the same operation to each element in a series, with the current computation depending on both the current input and the previous computations. **Plot 1** depicts a simple recurrent neural network, where the internal memory (h_t) is computed as:

$$h_t = g(Wx_t + Uh_t + b)$$

In this equation, $g()$ is the activation function (typically the hyperbolic tangent), U and W represent adjustable weight matrices for the hidden state (h), b denotes the bias term, and x is the input vector.

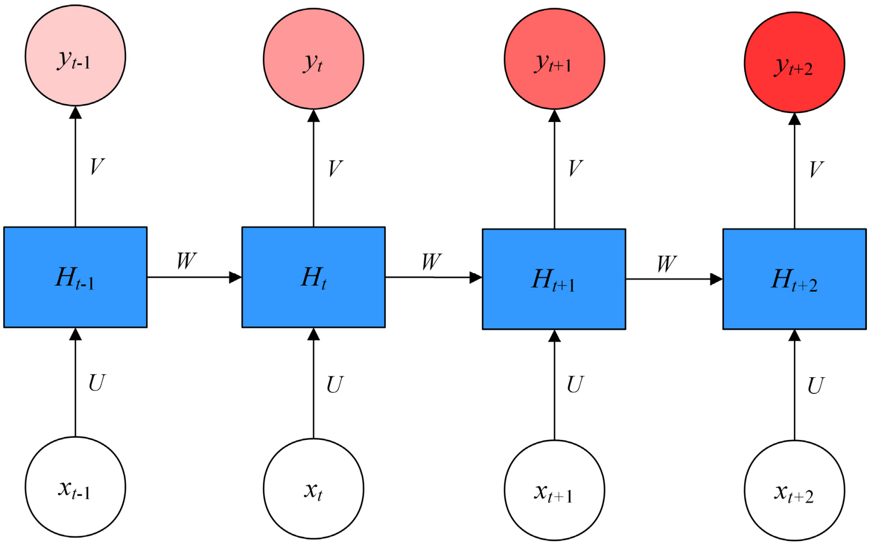

Plot 1: A simple recurrent neural network.

The advanced types of RNN models, such as LSTM and GRU are described as follows.

LSTM

LSTM network is a mature and excellent network for time series processing, which can capture the spatial-temporal correlation in time series prediction. Essentially, LSTM is a recurrent neural network for processing time series data. LSTM model solves the gradient disappearance problem of RNNs by

learning the long-term dependencies between time series. In practice, LSTM replaces the hidden layer of RNNs with a memory unit, which is controlled by a forget gate, an input gate and an output gate (see **Plot 2**). By using this memory unit, it can automatically select the best lag time and selectively remember historical information, thus solving the gradient disappearance problem caused by the long sequence of RNN.

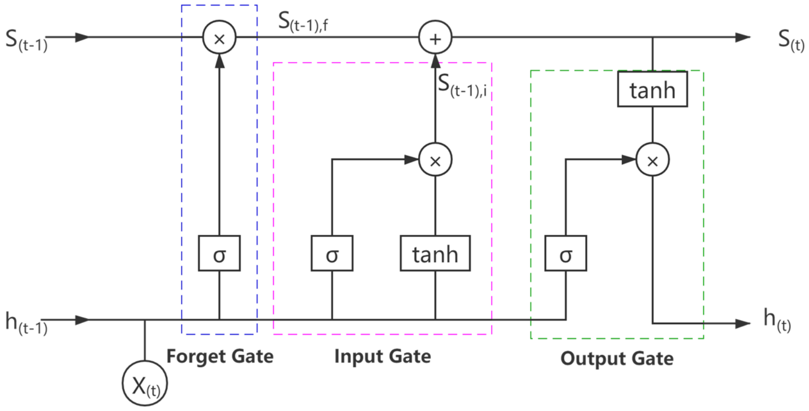

Plot 2: LSTM single structure diagram.

GRU

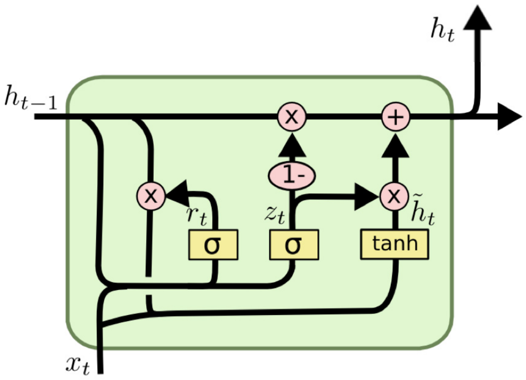

Plot 3: The structure of a GRU unit.

GRU is another variant of the RNN architecture that addresses the short-term memory issue and offers a simpler structure compared to LSTM. GRU combines the input gate and forget gate of LSTM into a single update gate, resulting in a more streamlined design. Unlike LSTM, GRU does not include a separate cell state. A GRU unit consists of three main components: an update gate, a reset gate, and the

current memory content. These gates enable the GRU to selectively update and utilize information from previous time steps, allowing it to capture long-term dependencies in sequences. **Plot 3** illustrates the structure of a GRU unit.

Supporting Material FILE S3: TCN and TCN-LSTM networks.

TCN

TCN is a new network that combines CNN and RNN structures to solve time series problems. TCN can receive an input sequence of arbitrary length as input and simultaneously map it to an output sequence of equal length. There is a causal relationship between the layers of the convolutional network and there is no 'missing' historical information or future data. In addition, it uses a combination of residual modules and extended convolution to create a very long effective history scale. Initial experimental evaluation of the TCN shows that the TCN architecture exhibits better performance than traditional recurrent networks such as LSTM on a variety of tasks and datasets, while also having a longer effective memory.

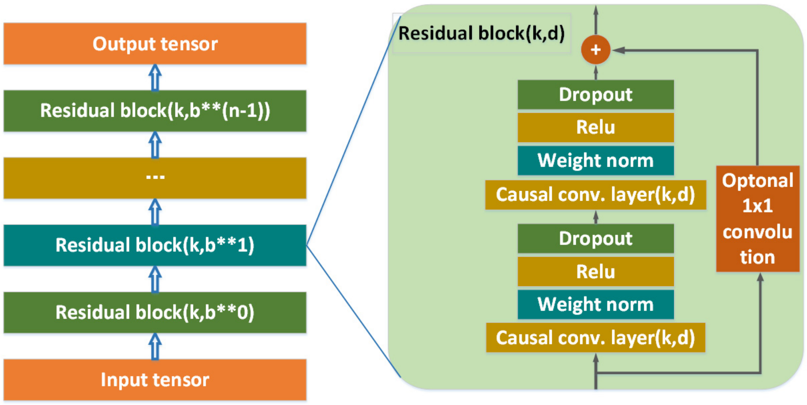

Plot 1: TCN structure diagram.

The TCN architecture has three main components: causal convolution, inflationary convolution and residual modules. Causal convolution is good at preserving previous historical information and prevents future information from being compromised. To effectively deal with the problem of long history information, the TCN architecture uses inflated convolution to increase the perceptual field. To cope with this, the network uses a result similar to the ResNet residual block, replacing the simple connection between TCN layers with a residual structure to make the TCN structure more generalizable. Considering that a deeper network structure may cause problems such as gradient disappearance, the application of

one-dimensional convolution ensures that the convolution kernel only moves along the temporal dimension. The structure of the TCN network is shown in **Plot 1**.

TCN-LSTM

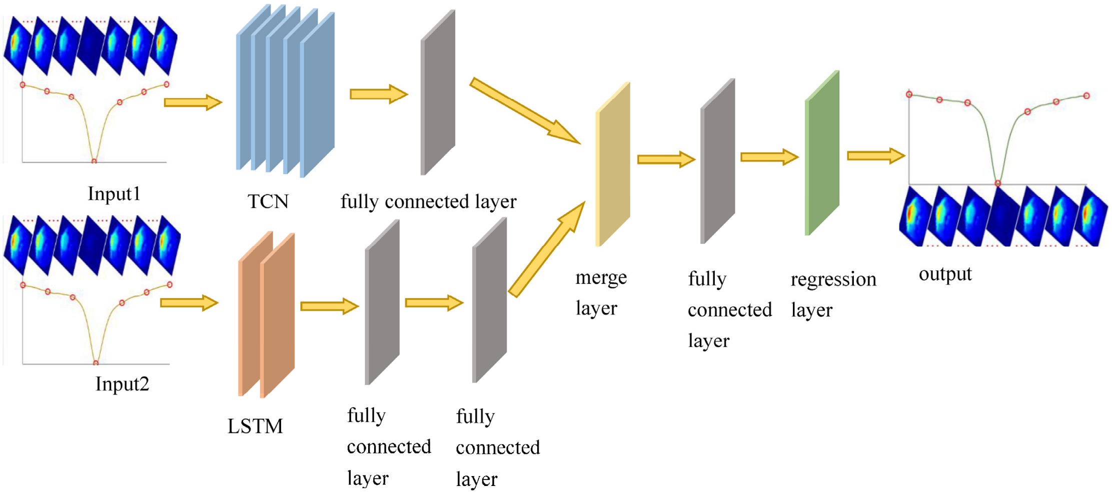

Plot 2: TCN-LSTM structure diagram.

Both LSTM and TCN have different methods and processes for processing time series problems, which leads to their respective characteristics. Bai S et al. showed that TCN is not always better than LSTM, and in some tasks TCNs perform better, while in other tasks LSTMs may be more effective. Hu et al. also found that TCNs have low prediction accuracy on short time scales and high prediction accuracy on long time scales, while the opposite is true in LSTMs. The inflated causal convolution structure allows TCN to have excellent feature extraction capability, which can fuse raw features to obtain high-dimensional abstract features and enhance the mining of feature information to extract long-term temporal relationships and higher-level spatial features from historical data, while LSTM has advantages in non-linear fitting and time-series prediction to capture dependencies from time-series data. By combining TCN and LSTM, where the LSTM remembers connections in the short term of the data and corrects TCN results for short term errors, the TCN-LSTM model can learn complex interactions of time series more effectively. Considering that the combination of TCN and LSTM may have better training

effect, we built a model based on the joint TCN-LSTM for prediction of CEST sequences to have better prediction effect. the structure diagram of the TCN-LSTM network model is shown in **Plot 2**.

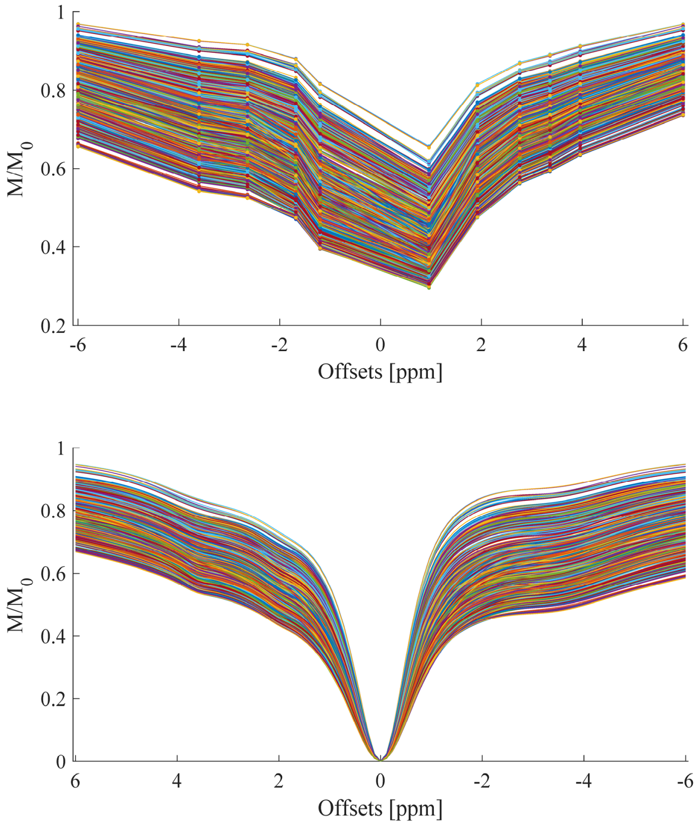

Supporting Material FIGURE S1: The visualization of simulated Z-spectra. The top plot denotes the Z-spectra at frequency offsets [-6.00, -3.60, -2.64, -1.68, -1.20, 0.96, 1.92, 2.76, 3.36, 3.96, 6.00] ppm; the bottom plot is the Z-spectra with 101 frequency offsets evenly distributed from −6 to 6 ppm.

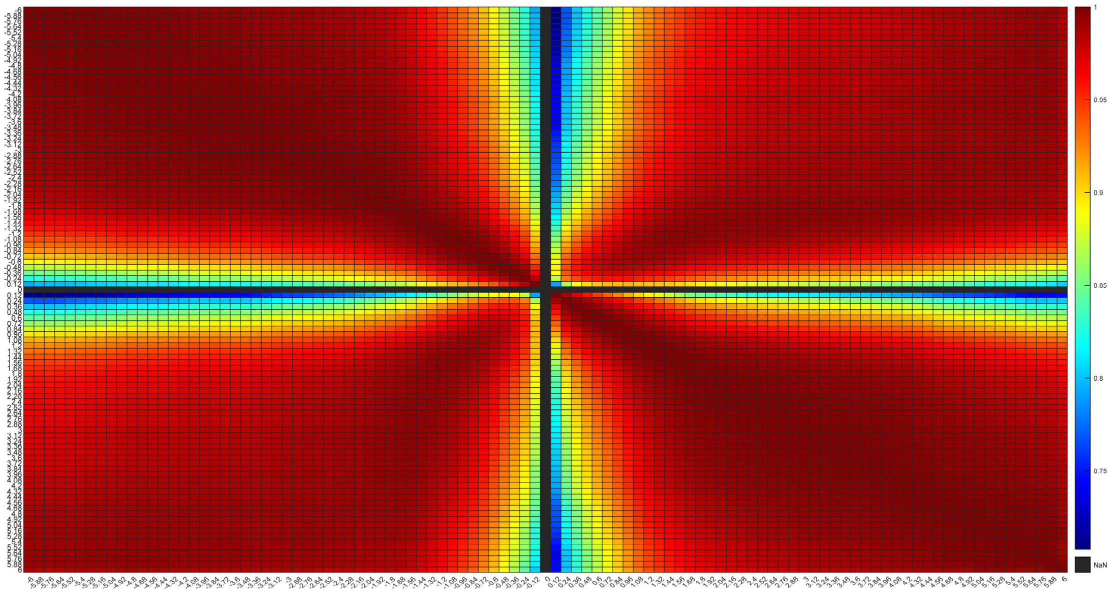

Supporting Material FIGURE S2: The heatmap of correlation coefficients matrix for experimentally acquired CEST images at frequency offsets [-6, 6] ppm.
